# Disrupting the Indian hedgehog signaling pathway *in vivo* attenuates surgically induced osteoarthritis progression in *Col2a1-CreER*^*T2*^; Ihh^fl^/^fl^ mice

**DOI:** 10.1186/ar4437

**Published:** 2014-01-15

**Authors:** Jingming Zhou, Qian Chen, Beate Lanske, Braden C Fleming, Richard Terek, Xiaochun Wei, Ge Zhang, Shaowei Wang, Kai Li, Lei Wei

**Affiliations:** 1 Department of Orthopedics, Warren Alpert Medical School, Brown University, Providence, RI 02903, USA; 2 Department of Oral Medicine, Infection, and Immunity, Harvard School of Dental Medicine, Boston, MA 02115, USA; 3 Department of Orthopedics, The Second Hospital of Shanxi Medical University, Taiyuan, Shanxi 030001, People’s Republic of China; 4 Hong Kong Baptist University, Baptist University Road, Kowloon Tong, Kowloon, Hong Kong SAR, People’s Republic of China

## Abstract

**Introduction:**

Previous observations implicate Indian hedgehog (Ihh) signaling in osteoarthritis (OA) development because it regulates chondrocyte hypertrophy and matrix metallopeptidase 13 (MMP-13) expression. However, there is no direct genetic evidence for the role of Ihh in OA, because mice with cartilage or other tissue-specific deletion of the *Ihh* gene die shortly after birth. We evaluated the role of Ihh *in vivo* via a Cre-loxP-mediated approach to circumvent the early death caused by *Ihh* deficiency.

**Methods:**

To evaluate the role of Ihh in OA development, *Ihh* was specifically deleted in murine cartilage using an *Ihh* conditional deletion construct (*Col2a1-CreER*^*T2*^; Ihh^fl/fl^). The extent of cartilage degradation and OA progression after *Ihh* deletion was assessed by histological analysis, immunohistochemistry, real-time PCR and *in vivo* fluorescence molecular tomography (FMT) 2 months after OA was induced by partial medial meniscectomy. The effect of Ihh signaling on cartilage was compared between *Ihh*-deleted mice and their control littermates.

**Results:**

Only mild OA changes were observed in *Ihh*-deleted mice, while control mice displayed significantly more cartilage damage. Typical OA markers such as type X collagen and MMP-13 were decreased in *Ihh*-deleted mice. *In vivo* FMT demonstrated decreased cathepsins and MMP activity in knee joints of animals with deletion of *Ihh*.

**Conclusions:**

These findings support the protective role of *Ihh* deletion in surgically induced OA. Thus, our findings suggest the potential to develop new therapeutic strategies that can prevent and treat OA by inhibiting Ihh signaling in chondrocytes.

## Introduction

Osteoarthritis (OA) is an extremely common disease that is characterized by progressive degeneration of articular cartilage and causes chronic joint pain and disability [1]. It has been reported that aging, trauma, excessive mechanical load and genetic defects are associated with OA development [2-4], but the exact signaling pathways involved in cartilage degeneration remain unclear. Recent evidence suggests that a bioactive protein, Indian hedgehog (Ihh), may be involved because blocking hedgehog (Hh) signaling with an inhibitor attenuated OA progression [5]. In mammals, the Hh family consists of three homologues: Ihh, Sonic hedgehog and Desert hedgehog, which all share the same signaling pathway. Ihh is a key signaling molecule and is synthesized and expressed primarily in prehypertrophic chondrocytes during growth plate development. The main function of Ihh is to regulate chondrocyte hypertrophy and thereby endochondral ossification [6-8]. It has been reported that some of the differentiation processes that occur during embryogenesis are also present in OA chondrocytes [9-13]. During endochondral ossification, cartilage degradation is induced by matrix metallopeptidases (MMPs), which are expressed by hypertrophic chondrocytes. Similar to this process, cartilage degradation in OA is mediated by MMPs [14-16]. Our interest in the present study was to evaluate the role of Ihh in OA development by specifically knocking down Ihh in chondrocytes to obtain more direct evidence that Ihh signaling is critical to OA progression.

Genetic studies using knockout mice have demonstrated that activation of Ihh downstream signaling pathways correlates with loss of articular cartilage thickness and proteoglycan (PG) content [7]. Consistent with these observations, increased Hh signaling is involved in mouse OA development [5] and increased type X collagen expression has been found in human knee joint cartilage with early, focal, OA-like lesions [11,17]. However, these studies did not exclude the possibility that other Hh members are also involved OA cartilage degeneration, nor did they establish which cells are involved in Hh signaling. Our previous study in which human OA tissues were analyzed for Ihh and hypertrophic marker contents, as well as the effect of Ihh signaling on OA chondrocyte hypertrophy, provided strong evidence that Ihh signaling may promote OA development by driving chondrocyte hypertrophy [18].

The role of the Hh pathway in postnatal skeletal homeostasis, however, is still unclear. Ohba *et al*. [19] and Mak *et al*. [20] reported conflicting findings. They used genetic mouse models to delete Patched, the membrane-bound downstream receptor of Hh that acts as a functional inhibitor of Hh signaling, to address the role of the enhanced Hh signaling in postnatal bone cells. Ohba *et al*. demonstrated that enhanced Hh signaling increased bone mass, whereas Mak *et al*. showed that enhanced Hh signaling resulted in decreased bone mass. Furthermore, similar observations have also been found in chondrocytes. Ihh inhibits chondrocyte hypertrophy indirectly by regulating parathyroid hormone–related protein (PTHrP) expression [21], but Ihh signaling can also directly promote chondrocyte hypertrophy in the absence of PTHrP [22]. The direct and indirect effects of Ihh signaling oppose each other. These context-dependent effects indicate that the mediation of cartilage changes in OA may be a complex network involving multiple factors. Therefore, it is important to obtain direct genetic evidence to demonstrate the role of Ihh in OA. Conventional Ihh-knockout mice are embryonic lethal, and even mice with cartilage-specific deletion of the *Ihh* gene (targeted by *Col2a1*-*Cre*) die shortly after birth [23]. To explore the role of Ihh in OA, we used a cartilage-specific, inducible loss of function approach. We used an OA model utilizing Ihh conditional knockout mice (*Col2a1*-*CreER*^*T2*^; *Ihh*^*fl/fl*^), a tamoxifen (TM)-inducible CreER^T2^ recombinase and partial medial meniscectomy (PMM) [24,25]. This murine model allowed us to test the hypothesis that inhibition of Ihh signaling decreases cartilage degeneration. A unique element of this assessment is the use of fluorescence molecular tomography (FMT) to monitor cathepsin and MMP activity in knee joints. Furthermore, we tested the effect of blocking Ihh in human cartilage explants to determine if the mouse results could be generalized to human tissue. The clinical implication of our study is that targeting Ihh signaling may be a viable strategy to prevent or treat OA.

## Methods

### Animals

*Col2a1-CreER*^*T2*^; Ihh^fl/fl^ mice (supplied by BL) were bred as previously described [6]. Two-month-old male *Col2a1-CreER*^*T2*^; Ihh^fl/fl^ mice (*N* = 60) were divided into two groups: TM (*n* = 30) and no TM (*n* = 30). Each group was subdivided into three subgroups: PMM (*n* = 12), sham (*n* = 10) and no surgery (*n* = 8). In the TM group, 2-month-old mice were injected with TM (1 mg/10 g/day for 5 consecutive days) to delete Ihh. In the no TM group mice, were injected with five doses of solvent (corn oil) as a control. The animals were randomized to undergo PMM surgery (the PMM group), sham surgery (the sham group) or no treatment (the no surgery group) at 3 months of age. They were killed at 5 months of age. Right hindlimbs were harvested immediately after the mice were killed. Approval of the animal experiments was obtained from the Institutional Animal Care and Use Committee at Rhode Island Hospital.

### Surgery

To induce posttraumatic OA in the PMM subgroups, the right anterior medial menisci were removed using a surgical microscope and a microsurgical technique as previously described [26]. After irrigating the surgical site with saline to remove tissue debris, the joint capsule and skin incision were closed in layers. During the procedure, close attention was paid not to injure the articular cartilage. The right hind knee joints of mice in the sham subgroups were sham-operated using the same approach, but without meniscectomy. Postoperatively, animals were allowed unrestricted activity and free access to food and water.

### Fluorescence molecular tomography

Inflammation-associated factors were monitored by FMT *in vivo* after 2 months. FMT enables real-time three-dimensional quantitation of fluorochrome distribution in tissues of live animals [27-30]. The mice were injected with a single dose of ProSense 750 EX and MMPSense 680 fluorescent imaging agents (PerkinElmer, Waltham, MA, USA) 24 hours before imaging. ProSense detects cathepsins B, L, and S and plasmin. MMPSense detects MMP-2, MMP-3, MMP-9 and MMP-13 activity. Mice were anesthetized with an intraperitoneal injection of ketamine (75 mg/kg) and medetomidine (1 mg/kg), placed in an upright position in the imaging chamber and then imaged using the VisEn FMT Optical Imaging System (PerkinElmer). A near-infrared laser diode emitting continuous wave radiation at wavelengths of 670 nm or 746 nm transilluminated the lower body of each animal from posterior to anterior, and both excitation and emission signals were detected by a charge-coupled device camera and appropriate band-pass filters. The no TM-PMM group (*n* = 12), TM-PMM group (*n* = 8) and TM-sham group (*n* = 3) were imaged. The picomolar concentrations of probes in the knee joint were determined using region of interest analysis. We used FMT imaging to confirm that Ihh deletion decreases inflammatory mediators *in vivo*, then compared the results with histological scores and molecular studies.

### Histology

After the mice were killed with carbon dioxide, the knee joints of right hindlimbs were harvested and immersed in 10% formalin for 72 hours. The specimens were decalcified in 20% ethylenediaminetetraacetic acid solution (pH 7.2). They were processed in a Tissue-Tek VIP 1000 tissue processor (Miles Laboratories, Elkhart, IN, USA) and embedded in a single block of Paraplast X-TRA medium (Sigma-Aldrich, St Louis, MO, USA). Blocks were trimmed to expose tissue using a rotary microtome (Reichert-Jung, Vienna, Austria), and 6-μm coronal sections were mounted on slides. Safranin O staining was performed, and the severity of cartilage damage was assessed using the Osteoarthritis Research Society International Osteoarthritis Cartilage Histopathology Assessment System (OOCHAS) (OA score = grade × stage; range, 0 to 24) [31]. Three independent blinded observers (LW, HL and JZ) scored each section, and the scores for the medial and lateral tibial condyles were averaged within each joint.

### Immunohistochemistry

To detect the distribution of Ihh, MMP-13 and types II and X collagens in cartilage, 6-μm sections were collected on positively charged glass slides (Thermo Fisher Scientific, Asheville, NC, USA). The sections were dried on a hotplate to increase adherence to the slides. Immunohistochemistry was carried out using the 3,3′-diaminobenzidine (DAB) streptavidin-peroxidase (SP) DAB Histostain-SP immunohistochemistry kit (ZYMED Laboratories/Invitrogen, Carlsbad, CA, USA). Sections were deparaffinized and rehydrated using conventional methods. Endogenous peroxidase was blocked by treating the sections with 3% hydrogen peroxide in methanol (Sigma-Aldrich) for 30 minutes. The sections were digested by 5 mg/ml hyaluronidase in phosphate-buffered saline (PBS) (Sigma-Aldrich) for 20 minutes. The sections were incubated with specific antibodies against Ihh (Santa Cruz Biotechnology, Santa Cruz, CA, USA), MMP-13 (Santa Cruz Biotechnology), types II and X collagens (Developmental Studies Hybridoma Bank, University of Iowa, Iowa City, IA, USA) and type II collagen breakdown product (IBEX Technologies, Mont-Royal, QC, Canada), respectively, at 4°C overnight. The negative control sections were incubated with isotype-matched control serum (2 μg/ml) (R&D Systems, Minneapolis, MN, USA) in PBS. Thereafter the sections were treated sequentially with biotinylated secondary antibody and SP conjugate (ZYMED Laboratories/Invitrogen), then developed in DAB chromogen (ZYMED Laboratories/Invitrogen). The sections were counterstained with hematoxylin (ZYMED Laboratories/Invitrogen). Photomicrographs were taken with a Nikon E800 microscope (Nikon, Melville, NY, USA) [32].

### Laser capture and real-time PCR

To quantify the mRNA levels of Ihh, *Gli1*, types II and X collagens, MMP-13, Runx2 and aggrecan, a formalin-fixed, paraffin-embedded Paradise PLUS Reagent System (MDS Analytical Technologeis/Molecular Devices, CA, USA) was used to extract and amplify RNA from articular cartilage [33]. Tissue sections (10 μm) were air-dried and dehydrated through graded alcohols and subjected to laser-captured microdissection (LCM) within 2 hours of deparaffinization as previously described [34]. Approximately 2,000 cells were present in microdissected articular cartilage. The articular cartilage was captured on LCM Macro CapSure Caps (Applied Biosystems) using the Arcturus AutoPix Automated Laser Capture Microdissection System (Applied Biosystems). The quantification of mRNA was performed by real-time PCR using the QuantiTect SYBR Green PCR kit (QIAGEN, Valencia, CA, USA) with the CFX384 Real-Time PCR Detection System (Bio-Rad Laboratories, Hercules, CA, USA). Each reaction was performed in triplicate. The following primers were used: mouse Ihh: forward, 5′-CCA CTT CCG GGC CAC ATT TG-3′, and reverse, 5′-GGC CAC CAC ATC CTC CAC CA-3′; mouse *Gli1*: forward, 5′-GGT CCG GAT GCC CAC GTG AC-3′, and reverse, 5′-TCC CGC TTG GGC TCC ACT GT-3′; mouse Gli2: forward, 5′-TGG CAG CGA TGG GCC TAC CT-3′, and reverse, 5′-GCC GTG TGC TGC TGT TTG GC-3′; mouse type X collagen: forward, 5′-GCC AGG AAA GCT GCC CCA CG-3′, and reverse, 5′-GAG GTC CGG TTG GGC CTG GT-3′; mouse MMP-13: forward, 5′-GGA CCT TCT GGT CTT CTG GC-3′, and reverse, 5′-GGA TGC TTA GGG TTG GGG TC-3′; mouse Runx2: forward, 5′-CCG CAC GCA AAC CGC ACC AT-3′, and reverse, 5′-CGC TCC GGC CCA CAA ATC TC-3′; and mouse aggrecan: forward, 5′-CAG TGG GAT GCA GGC TGG CT-3′, and reverse, 5′-CCT CCG GCA CTC GTT GGC TG-3′. Amplification conditions were as follows: 2-minute preincubation at 50°C; 10 minutes at 95°C for enzyme activation; and 40 cycles at 95°C denaturation for 10 seconds, 55°C annealing for 30 seconds and 72°C extension for 30 seconds. The comparative threshold cycle (Ct) method, that is, the 2^−ΔΔCt^ method, was used to calculate fold amplification [34].

### Human articular cartilage organ culture and real-time PCR

Human OA cartilage samples were obtained during knee replacement surgery. This part of the study was also approved by the Institutional Review Board at Rhode Island Hospital, and informed consent was obtained from each donor. The samples were cut into 4-mm^3^ pieces, cultured in Dulbecco’s modified Eagle’s medium containing 10% fetal bovine serum and treated with the Hh inhibitor cyclopamine (20 μM) or dimethyl sulfoxide (DMSO) as the control. Total RNA was isolated after 48 hours of treatment. The quantification of mRNA was performed by real-time PCR using the QuantiTect SYBR Green PCR kit with the CFX384 Real-Time PCR Detection System as described above. The following primers were used: human *Gli1*: forward, 5′- GAA CCC TTG GAA GGT GAT ATG TC-3′, and reverse, 5′-GGC AGT CAG TTT CAT ACA CAG AT-3′; human type X collagen: forward, 5′-TGC CTC TTG TCA GTG CTA ACC-3′, and reverse, 5′-GCG TGC CGT TCT TAT ACA GG-3′; and human MMP-13: forward, 5′-TGC TGC ATT CTC CTT CAG GA-3′, and reverse, 5′- ATG CAT CCA GGG GTC CTG GC-3′. Gene expression levels were calculated as described above.

### Statistical analysis

Data are expressed as means ± SD. Two-tailed paired *t*-tests were used to compare mRNA levels between the no TM-PMM and TM-PMM groups, and between the cyclopamine and DMSO groups. MMPSense and ProSense signals between the no TM-PMM and TM-PMM groups were also compared using two-tailed, paired *t*-tests. A probability level less than 5% was considered significant. The OOCHAS score in different groups were analyzed by one-way analysis of variance with multiple pair-wise comparisons made by the Student-Newman-Keuls method (three comparisons or more) at a rejection level of 5% unless otherwise noted.

## Results

### Validation of animal model

**Figure 1:**
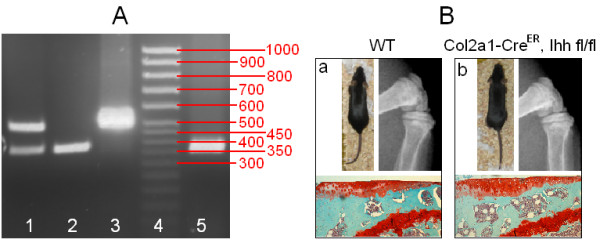
***Col2a1-CreER***^***T2***^**; Ihh**^**fl/fl **^**mice exhibit normal phenotype before induction. (A)** Insertion of loxP restriction enzyme site in the *Indian hedgehog* (*Ihh*) allele was confirmed by PCR. Lane 1: heterozygous *Ihh*^*fl/fl*^ (382 bp and 484 bp), lane 2: homozygous *Ihh*^*fl/fl*^ (382 bp), lane 3: wild type (WT) (484 bp), lane 4: 50-bp DNA marker and lane 5: Cre-recombinase (328 bp). **(B)** X-rays and Safranin O–stained images show normal growth plate and proteoglycan staining in WT mice **(a)** and mutant mice **(b)** at 2 months of age without deletion of *Ihh*.

**Figure 2:**
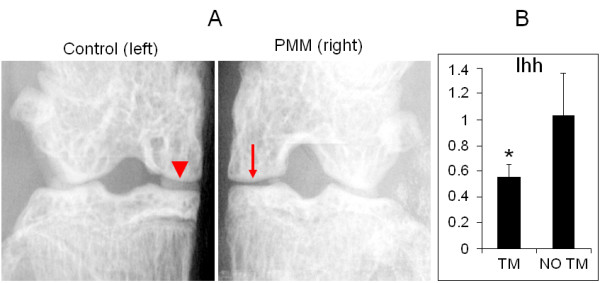
**Validation of Indian hedgehog deletion and partial medial meniscectomy. (A)** Radiographs using soft-tissue settings show medial meniscus of the left knee joint without partial medial meniscectomy (PMM) surgery (indicated by arrowhead in left panel) and absence of medial meniscus of the right knee joint (indicated by arrow in right panel). **(B)** Results of real-time PCR are shown. Relative fold expression of Indian hedgehog (Ihh) is decreased by 46.5% in tamoxifen (TM)-injected mice. Data are means ± SD. TM, *n* = 6. no TM, *n* = 8. *P < 0.005.

Several research groups have used the *Col2a1-CreER*^*T2*^*; Ihh*^*fl/fl*^-transgenic mouse model, which they validated in their published papers [35-37]. In our present study, PCR was used for genotyping to confirm disruption of both *Ihh* alleles and the presence of Cre (Figure 1A). Before TM induction, homozygous transgenic and wild-type mice showed no difference in phenotypes, that is, similar body size, normal PG staining and open growth plates, at 2 months of age (Figure 1B). One month after TM injection, PMM surgery was performed on the right knees. Radiographic analysis was used to confirm that right medial menisci had been removed successfully (Figure 2A). Our real-time PCR results showed knockdown of Ihh expression in cartilage 3 months after TM injection (Figure 2B). Closure of the growth plate was also consistently evidenced after TM was injected (see details in Additional file 1). These observations are in accord with previous findings in which Cre-recombinase-mediated deletion of a floxed gene expressed in articular chondrocytes at the adult stage can be highly specific and retain high efficiency several months after induction [38,39].

### Deletion of Ihh has chondroprotective effects

**Figure 3:**
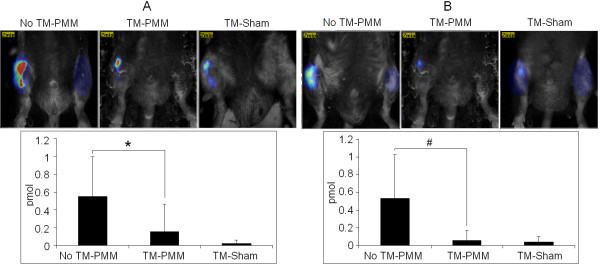
**Cathepsin and matrix metalloprotease activity were decreased in Indian hedgehog–deleted mice 2 months after partial medial meniscectomy.** Representative images produced with ProSense **(A)** and MMPSense **(B)** fluorescent imaging agents (top panels) and summary results (bottom panels) are shown. Data are means ± SD. No tamoxifen–partial medial meniscectomy (TM-PMM), *n* = 12; TM-PMM, *n* = 8; and TM-sham, *n* = 3. *P = 0.016, #*P* = 0.042.

**Figure 4:**
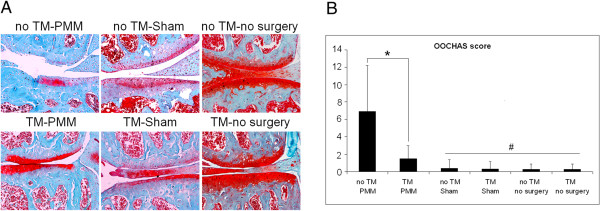
**Deletion of Indian hedgehog alleviates osteoarthritis cartilage damage. (A)** Coronal sections of mouse knee joints are shown after Safranin O staining. PMM, partial medial meniscectomy; TM, tamoxifen. **(B)** Osteoarthritis Research Society International Osteoarthritis Cartilage Histopathology Assessment System (OOCHAS) scores are shown. Data are means ± SD. No TM-PMM, *n* = 12; TM-PMM, *n* = 12; TM-Sham, *n* = 10. *P < 0.0001. In the control groups (No TM-Sham, *n* = 10; TM-Sham, *n* = 10; No TM-No Surgery, *n* = 8; TM-No Surgery, *n* = 8), there were no significant differences, #*P* = 0.585.

Mice in the no TM-PMM group had more signaling detected by ProSense and MMPSense fluorescent imaging agent in the surgical knee compared to the TM-sham group, indicating that PMM surgery elevated protease activity in the knee joint (Figure 3). Deletion of Ihh reduced the ProSense signal in the TM-PMM mice by 89.0% compared to control mice (no TM-PMM) (*P* = 0.016) (Figure 3A). MMPSense signal in the TM-PMM group was decreased by 71.9% compared to the no TM-PMM group (*P* = 0.042) (Figure 3B). Overall, the FMT results show that deletion of Ihh leads to decreased production of proteases after surgery, which is in accordance with less severe OA damage in the TM-PMM mice (Figure 4).

Histological analysis demonstrated that the articular cartilage surface of the knee joint in the TM-PMM group was Safranin O–positive with a relatively intact cartilage surface. In contrast, the no TM-PMM group, in which Ihh was present, exhibited severe cartilage damage and loss of PG staining (Figure 4A). We also quantified the extent of OA damage using the OOCHAS score. The entire tibial cartilage surface of each sample was scored by three independent reviewers. In the no TM-PMM group, cartilage displayed more severe OA damage than the TM-PMM group, in which Ihh was knocked down (Figure 4B). Consistent with the Safranin O staining results, the OOCHAS scores of the sham groups and the no-surgery groups were close to zero, indicating minimal OA damage (Figure 4B). The TM-no Surgery and no TM–no Surgery groups showed no change in OOCHAS scores (Figures 4A and 4B), which excluded the possibility that TM had an effect on cartilage. We also compared the TM-sham and no TM–sham groups. As expected, the sham operation did not induce OA damage in cartilage (Figures 4A and 4B). The TM-sham group was used as the control group in the next experiments.

**Figure 5:**
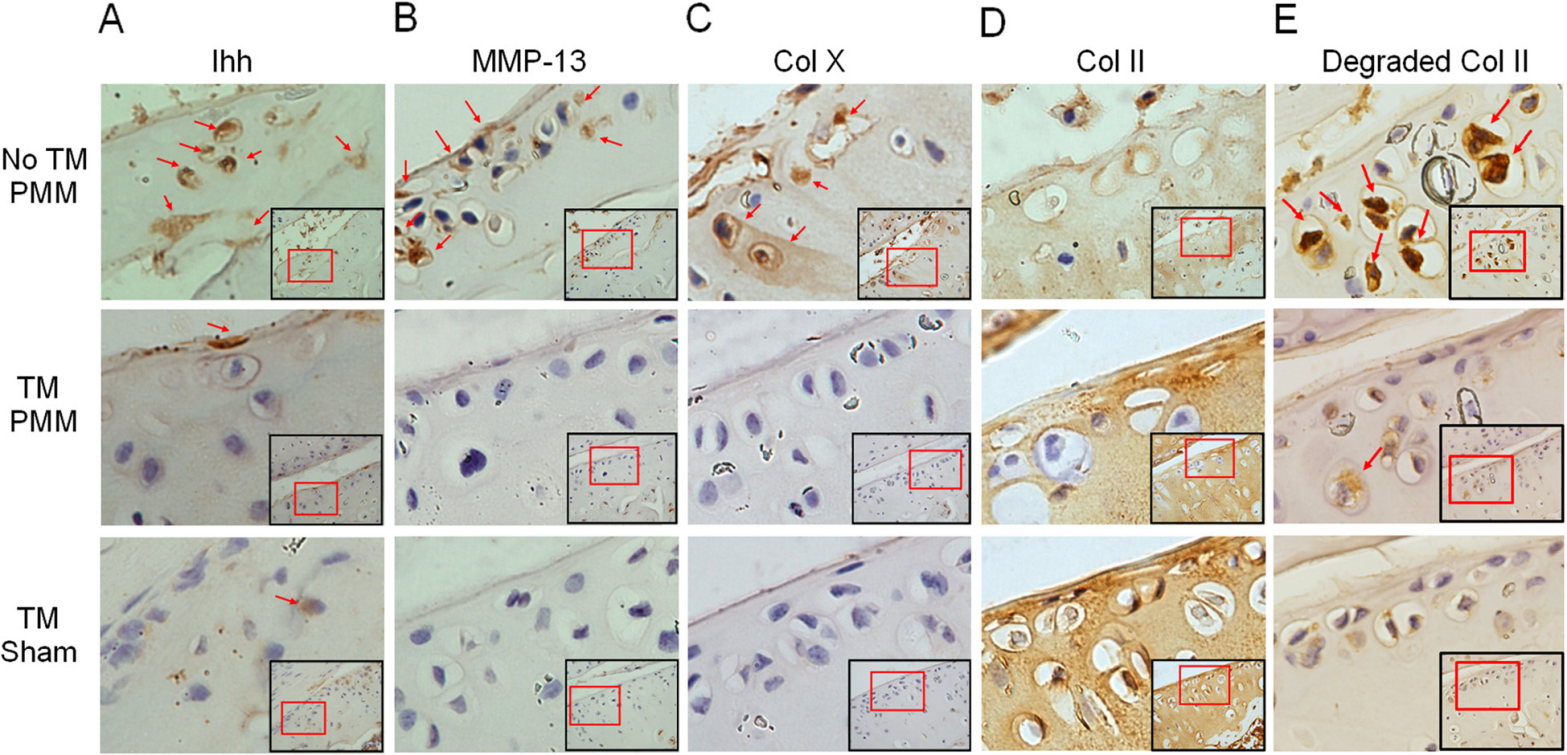
**Indian hedgehog, matrix metalloprotease 13 and type X collagen expression is reduced in Ihh-deleted mice, but type II collagen and aggrecan are preserved.** Immunohistochemical staining was used in osteoarthritis (OA)-induced tibial articular cartilage of 5-month-old mice for Indian hedgehog (Ihh) **(A)**, matrix metalloprotease 13 (MMP-13) **(B)**, type X collagen (Col X) **(C)**, type II collagen (Col II) **(D)** and type II collagen breakdown products (Degraded Col II) **(E)**. PMM, partial medial meniscectomy; TM, tamoxifen. Positive signals (brown staining) are indicated by arrows. Inside the black box was image taken under the 20x objective lens, and from which a small area was further enlarged under 20x objective lens (inside the red box)

Immunohistochemistry was performed to determine the expression of MMPs and type X collagen. In the TM-PMM and TM-sham groups, Ihh expression in chondrocytes was less than in the no TM–PMM group (Figure 5A). MMP-13 and type X collagen were elevated in cartilage of the no TM–PMM group compared to Ihh-deleted cartilage (the TM-PMM and TM-sham groups), in which less OA damage was observed (Figures 5B and 5C). In contrast, type II collagen content was higher in cartilage in the TM-PMM and TM-sham groups than in the no TM–PMM group (Figure 5D), and type II collagen degradation was inhibited in the TM groups compared to the no TM–PMM group (Figure 5E).

**Figure 6:**
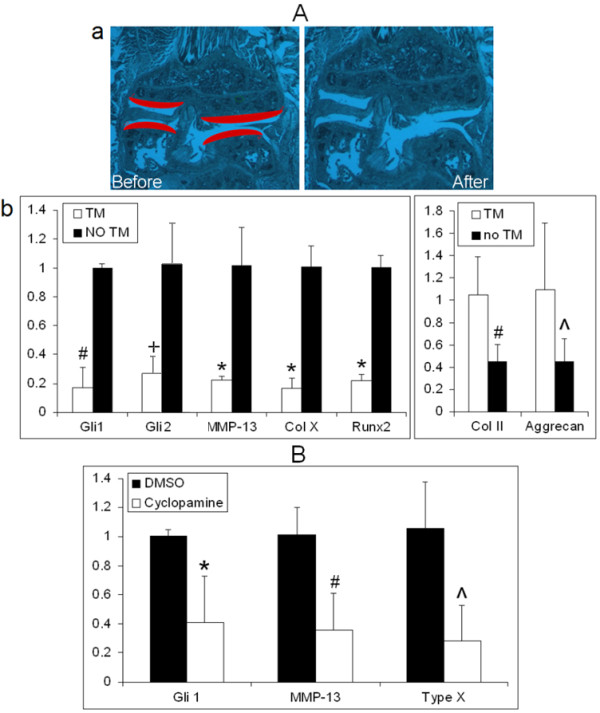
**Decreased Indian hedgehog signaling inhibits Ihh downstream targets in osteoarthritis cartilage. (A)** Indian hedgehog (Ihh) deletion leads to downregulation of Ihh downstream targets in osteoarthritis (OA) cartilage 2 months after partial medial meniscectomy. **(a)** Laser-captured microdissection was used to analyze gene expression in articular cartilage. Articular cartilage (highlighted in red) before and after microdissection is shown. **(b)** Results of real-time PCR are shown. The expression of Ihh, Gli1, Gli2, matrix metalloprotease (MMP-13), type X collagen (Col X) and Runx2 was significantly decreased, and the expression of aggrecan and type II collagen (Col II) was increased, respectively, in the Ihh-deleted mice (tamoxifen (TM)) (*n* = 6) compared to the control animals (no TM) (*n* = 8). Data are means ± SD. *P < 0.00001, #*P* < 0.0005, +*P* < 0.001, ^*P* < 0.05. **(B)** Cyclopamine decreased expression of *Gli1*, MMP-13 and type X collagen in human OA cartilage. Relative mRNA levels in human cartilage organ cultures 48 hours after treatment with cyclopamine (*n* = 8) and dimethyl sulfoxide (DMSO) controls (*n* = 8) are shown. Data are means ± SD (*n* = 8). *#^*P* < 0.0001.

Consistent with the immunohistochemical data (Figure 5), real-time PCR revealed a significant decrease in Ihh expression in Ihh-deleted mice (TM) compared to control mice (no TM). Similarly, in the Ihh-deleted mice, the expression of Gli1, Gli2, type X collagen, MMP-13 and Runx2 were decreased and aggrecan and type II collagen were increased (Figure 6A). Human cartilage organ culture also showed decreased Gli1, type X collagen and MMP-13 after treatment with the Hh inhibitor cyclopamine (Figure 6B).

## Discussion

The results of our study demonstrate that Ihh signaling is part of the pathobiology of OA development. Previous studies have demonstrated that Ihh plays an important role during growth plate and bone development by regulating type X collagen, MMP-13 and Runx2 expression [5,10,40-43]. In our previous study, we reported elevated levels of Ihh in human OA cartilage and synovial fluid compared to normal control samples [18]. Moreover, we earlier found that upregulation of Ihh promoted the hypertrophic phenotype and induced typical hypertrophic markers such as type X collagen and MMP-13 [18]. Therefore, upregulation of Ihh signaling may facilitate OA development by inducing chondrocyte hypertrophy and the expression of genes known to cause cartilage degeneration, confirming previous observations made by others [10,40,41,44]. Our findings are in agreement with those of Lin *et al*., who reported that human cartilage explants treated with Hh blocking agents exhibited decreased expression of type X collagen and MMP-13, but that Ihh ligand stimulation induced the expression of these two genes [5]. Thus, it is likely that induction of type X collagen and MMP-13 may be caused by increased Ihh signaling in OA cartilage *in vivo*. However, these previous studies were unable to exclude the possibility that other Hh family members are also involved OA cartilage degeneration or to determine whether Ihh signaling is associated with OA development, a secondary pathway or an attempt at healing damaged OA cartilage by reactivating developmental pathways.

In this study, we used, for the first time to the best of our knowledge, genetically deleted *Ihh* mice to directly study directly the role of Ihh in OA cartilage degeneration. Our results provide solid evidence that the deletion of *Ihh* prevents cartilage damage at the tissue level (Figures 4A and B). Gli2 and Gli3 are major signaling molecules in the Ihh pathway that promote osteoblast formation by regulating Runx2 [42]. As a direct downstream target of the Ihh pathway, Gli1 is regulated by Gli2 and Gli3 [45]. We also found that at the cellular and molecular levels, Gli1 and Gli2, type X collagen (the typical hypertrophic chondrocyte marker) and other cartilage-degrading enzymes, such as MMPs and cathepsins are effectively downregulated by Ihh deletion (Figures 3, 5 and 6A). In contrast, increased cartilage matrix molecules type II collagen and aggrecan were present in Ihh-deleted mice (Figures 5 and 6A). We further demonstrated that Ihh blockade by cyclopamine inhibits the expression of type X and MMP-13 in human cartilage organ cultures. Increased MMP-13 activity leads to structural damage in murine OA cartilage, and knockdown of MMP-13 decreases cartilage damage in OA [41]. Thus, our finding that deletion or inhibition of Ihh by genetic and pharmacological approaches downregulates MMP-13 expression and activity suggests that chondroprotection in patients with early-stage disease may be possible by inhibiting this pathway.

We have also demonstrated that FMT is highly consistent with the molecular and immunohistochemical results in our OA model (Figure 3). Therefore, FMT provides a new means by which to assess the effects of OA treatments on inflammation and cartilage degradation in the murine model *in vivo*. This noninvasive technique has been used for research in oncology as well as in inflammatory, pulmonary, cardiovascular and skeletal diseases [27-30]. The advantages of this technology are its capability to longitudinally monitor and quantify biological targets *in vivo* over multiple time points and to better understand the mechanism and progression of disease. Furthermore, FMT can be used to support data derived from other cellular and molecular *in vitro* assays. Peterson *et al*. compared FMT with traditional methods in evaluating rheumatoid arthritis. Their study showed that FMT provides more sensitive readouts in the pathology of the disease and that it is possible to use FMT results to predict disease development [30]. In our present study, we found that the findings obtained using the ProSense and MMPSense fluorescent imaging agents were consistent with our histological results. The value of FMT to study OA onset and progression is encouraging but still requires validation in future longitudinal studies.

Recent studies have demonstrated that Ihh expression is very low in healthy human articular cartilage but increases during OA development and that increased Ihh expression is associated with the severity of OA cartilage damage [18]. Furthermore, the increase of Ihh has been also reported in early-stage human articular cartilage lesions [17]. In our present study, OA changes were observed 2 months after surgery, which is enough time for OA to develop to a stage at which Ihh-deleted and nondeleted groups can be differentiated. Therefore, we did not evaluate shorter and longer time periods. However, previous reports on human OA cartilage tissues have suggested that the increase of Ihh may play a role in the initiation of OA [17,18].

Our findings suggest the therapeutic potential of targeting Ihh to prevent and treat OA cartilage degeneration. However, it is not possible to delete *Ihh* in larger animals, and *Ihh* gene deletion is not an option for OA treatment in humans. Chemical inhibitors of Hh signaling cause severe side effects, including holoprosencephaly, cleft lip and palate and limb defects [46-50]. Therefore, efficient knockdown of Ihh achieved by local delivery of small interfering RNA (siRNA) might be a more effective strategy. Recent studies have demonstrated that both liposome particles and nanoparticles can be used for local siRNA delivery to musculoskeletal tissues [51,52]. These techniques could be used to study the protective effects of Ihh knockdown in future studies utilizing large animal OA models.

## Conclusions

This study provides direct evidence that knockout of Ihh prevents the development of OA and that FMT can be used to evaluate cartilage health *in vivo* in the murine model. This chondroprotective effect results from inhibiting chondrocyte hypertrophy and the expression of genes known to cause cartilage degradation. Moreover, our results give further support to the concept of Ihh inhibition as a therapeutic strategy to prevent and treat OA.

## Abbreviations

FMT: Fluorescence molecular tomography; Hh: Hedgehog; Ihh: Indian hedgehog; MMP: Matrix metallopeptidase; OA: Osteoarthritis; OOCHAS: Osteoarthritis Research Society International Osteoarthritis Cartilage Histopathology Assessment System; PG: Proteoglycan; PMM: Partial medial meniscectomy; PTHrP: Parathyroid hormone-related protein; ROI: Region of interest; TM: Tamoxifen.

## Competing interests

The authors declare that they have no competing interests.

## Authors’ contributions

JZ and LW participated in study design, data acquisition, analysis and interpretation; manuscript preparation; and statistical analyses. SW and KL performed the PMM surgery and prepared the manuscript. XW, BL, RT, BCF, QC and GZ participated in study design, data analysis and interpretation and manuscript preparation. All authors read and approved the final version of the manuscript.

## Supplementary Material

Additional file 1 Growth plate closed after tamoxifen (TM) injection. Click here for file
