# Supplementary material for: Disrupting the Indian hedgehog signaling pathway in vivo attenuates surgically induced osteoarthritis progression in Col2a1-CreERT2; Ihhfl/fl mice
Source: Arthritis Res Ther. 2014 Jan 15;16(1):R11. doi: 10.1186/ar4437 (PMC3978435; doi:10.1186/ar4437)
Supplement: Additional file 1 — Growth plate closed after tamoxifen (TM) injection. [file ar4437-S1.pdf]

In this study, we have two directive evidences to show Ihh was efficiently knockdown: 1) the growth plate closure was evidenced when tamoxifen (TM) was admitted at 2-month-old animals but not in oil treated groups (supplemental Fig. 1); 2) Real time PCR indicated half of Ihh was knockdown in the TM animals compared to the oil control group (Fig. 2B). These directive evidences suggests that TM administration in 2-month-old animals could efficiently knockdown Ihh.

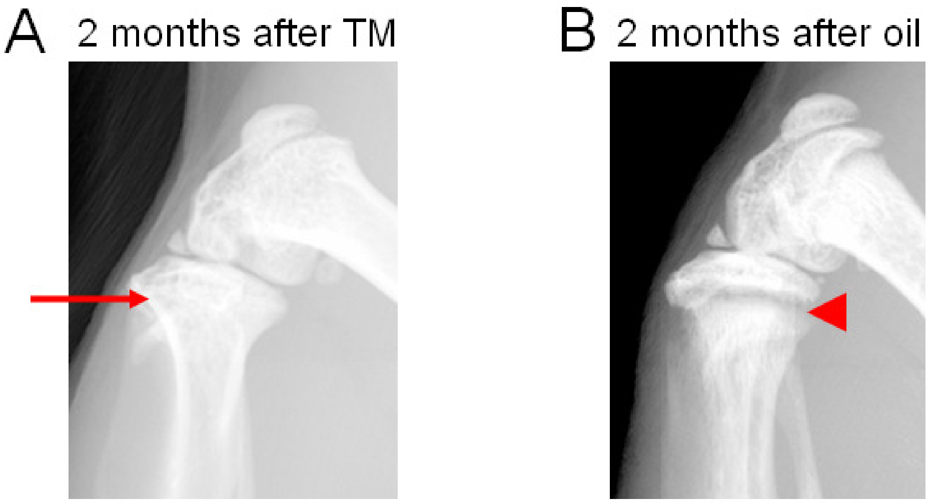

Suppl Fig 1. Growth plate closed after tamoxifen (TM) injection. (A) TM induced deletion of Ihh, resulting in growth plate closure (indicated by arrow). (B) Normal growth plate after oil injection is indicated by arrow head.
